# NADPH Oxidase as a Therapeutic Target for Oxalate Induced Injury in Kidneys

**DOI:** 10.1155/2013/462361

**Published:** 2013-06-06

**Authors:** Sunil Joshi, Ammon B. Peck, Saeed R. Khan

**Affiliations:** ^1^Department of Pathology, Immunology & Laboratory Medicine, University of Florida College of Medicine, Gainesville, FL 32610, USA; ^2^Department of Infectious Diseases & Pathology, University of Florida College of Veterinary Medicine, Gainesville, FL 32610, USA; ^3^Department of Urology, University of Florida College of Medicine, Gainesville, FL 32610, USA

## Abstract

A major role of the nicotinamide adenine dinucleotide phosphate (NADPH) oxidase family of enzymes is to catalyze the production of superoxides and other reactive oxygen species (ROS). These ROS, in turn, play a key role as messengers in cell signal transduction and cell cycling, but when they are produced in excess they can lead to oxidative stress (OS). Oxidative stress in the kidneys is now considered a major cause of renal injury and inflammation, giving rise to a variety of pathological disorders. In this review, we discuss the putative role of oxalate in producing oxidative stress via the production of reactive oxygen species by isoforms of NADPH oxidases expressed in different cellular locations of the kidneys. Most renal cells produce ROS, and recent data indicate a direct correlation between upregulated gene expressions of NADPH oxidase, ROS, and inflammation. Renal tissue expression of multiple NADPH oxidase isoforms most likely will impact the future use of different antioxidants and NADPH oxidase inhibitors to minimize OS and renal tissue injury in hyperoxaluria-induced kidney stone disease.

## 1. Introduction

In this review, we aim at focusing on the putative role of oxalate (C_2_O_4_^2−^) leading to oxidative stress (OS) by production of reactive oxygen species (ROS) via different isoforms of nicotinamide adenine dinucleotide phosphate (NADPH) oxidase present in the kidneys. First, we provide a background of different types of hyperoxaluria and address the factors involved in oxalate and calcium-oxalate (CaOx-) induced injury in the kidneys. Second, we aim at addressing the role and different types of ROS and other free radicals, which when overproduced lead to OS and a brief description of different markers in the kidney which increase during OS. Third, we discuss the different isoforms of NADPH oxidase, their location, function, and expression in different cell types. Fourth, we address the pathophysiological role of NADPH oxidase in the kidneys and the regulation of NADPH oxidase (NOX enzymes). Finally, we discuss the role of antioxidants used for renal treatment and the different NADPH oxidase inhibitors involved in blocking NADPH oxidase from catalyzing production of superoxide with a potential of reducing OS and injury in the kidneys.

Oxalate, the conjugate base of oxalic acid (C_2_H_2_O_4_), is a naturally occurring product of metabolism that at high concentrations can cause death in animals and less frequently in humans due to its corrosive effects on cells and tissues [1]. It is a common ingredient in plant foods, such as nuts, fruits, vegetables, grains, and legumes, and is present in the form of salts and esters [2–4]. Oxalate can combine with a variety of cations such as sodium, magnesium, potassium and calcium to form sodium oxalate, magnesium oxalate, potassium oxalate, and calcium oxalate, respectively. Of all the above oxalates, calcium oxalate is the most insoluble in water, whereas all others are reasonably soluble [5]. In normal proportions, it is harmlessly excreted from the body via the kidneys through glomerular filtration and secretion from the tubules [6, 7]. Oxalate, at higher concentrations, leads to various pathological disorders such as hyperoxaluria, nephrolithiasis (formation and accumulation of CaOx crystals in the kidney), and nephrocalcinosis (renal calcifications) [1, 5, 8, 9]. Hyperoxaluria is considered to be the major risk factor for CaOx type of stones [10] with nearly 75% of all kidney stones composed of CaOx [9]. These CaOx crystals, when formed, can be either excreted in the urine or retained in different parts of the urinary tract, leading to blockage of the renal tubules, injury to different kinds of cells in the glomerular, tubular and intestinal compartments of the kidney, and disruption of cellular functions that result in kidney injury and inflammation, decreased and impaired renal function [11, 12], and end-stage renal disease (ESRD) [13, 14]. Excessive excretion of oxalate in the urine is known as hyperoxaluria and a significant number of individuals with chronic hyperoxaluria often have CaOx kidney stones. Dependent on food intake, a normal healthy individual is expected to have a regular urinary oxalate excretion somewhere between 10–40 mg/24 h (0.1–0.45 mmol/24 h). Anything over 40–45 mg/24 h (0.45–0.5 mmol/24 h) is regarded as clinical hyperoxaluria [15, 16]. Hyperoxaluria can be commonly classified into three types: primary, secondary, and idiopathic. Primary hyperoxaluria in humans is generally due to a genetic defect caused by a mutation in a gene and can be further subdivided into three subgroups, type I–III. It is inherited in an autosomal recessive pattern and results in increased oxalate synthesis due to disorders of glyoxalate metabolism. There is inability to remove glyoxylate. Primary hyperoxaluria type I (PH I) is the most abundant of the three subgroups of primary hyperoxaluria (70–80%) [13], caused by the incorrect sorting of hepatic enzyme alanine-glyoxylate aminotransferase (AGT) to the endosomes instead of the peroxisomes. AGT function is dependent on pyridoxal phosphate protein and converts glyoxalate to glycine. Owing to deficiency of AGT in PH I cases, glyoxalate is alternatively reduced to glycolate and oxidized to oxalate. In some cases of PH I, AGT is present but is misdirected to mitochondria where it remains in an inactive state. The metabolic defect of PH I is restricted to liver peroxisomes and the AGT fails to detoxify glyoxalate in the peroxisomes. Primary hyperoxaluria type II (PH II) results from the scarcity of hepatic enzyme glyoxylate reductase/hydroxypyruvate reductase (GRHPR) activity normally found in the cytosol. In studies, different cohorts have shown concentrations of urinary oxalate excretion between 88–352 mg/24 h (1–4 mmol/24 h) for PH I and 88–176 mg/24 h (1-2 mmol/24 h) for PH II [13, 17, 18]. In some cases, there is natural occurrence of AGT and GRHPR activities, but still there may be PH type III due to anion exchanger SLC26A6 and mutations in DHDPSL [13, 19–21]. All three types of PH show symptoms from infant to adolescence stages, with a majority showing clinical symptoms at 5 years in PH I to 15 years in PH II, and during the neonatal years in PH III [18]. Approximately, 35% of patients with PH I may be unnoticed due to misinterpretation, lack or subtlety of the symptoms, until the onset of renal failure [13].

In contrast to primary hyperoxaluria, secondary hyperoxaluria appears to result from eating foods rich in high-oxalate levels or exposure to large amounts of oxalate/oxalate precursors. Regular daily oxalate consumption by Western populations varies highly from 44 to 351 mg/day (0.5–4 mmol/day) but may exceed 1000 mg/day (11.4 mmol/day) when oxalate rich foods (e.g., spinach or rhubarb) are eaten in excess [3, 22–24]. Exceedingly high values of up to 2045 mg/day have also been reported due to consumption of seasonal foods consisting of purslane, pigweed, amaranth, and spinach [25]. There are different factors that affect dietary oxalate absorption such as oxalate bioavailability in the gut after it is consumed, number and accessibility of cations that attach to oxalate, such as calcium (Ca^2+^) and magnesium (Mg^2+^) in the gut, oxalate precursors and their effect on dietary oxalate, inherited absorption capacity, emptying of the gastrointestinal fluids, time taken for transit in the intestine, and the accessibility of oxalate degrading microorganisms such as *Oxalobacter formigenes *[15, 22–26]. A further subtype of hyperoxaluria is idiopathic hyperoxaluria which is spontaneous with unknown causes. Previous research has shown that idiopathic CaOx stone patients have the ability to absorb a greater quantity of oxalate as compared to normal individuals [27–29]. This may be true for why some autistic children have a high state of hyperoxaluria.

Previous studies have shown that dietary oxalate usually contributes just 10–20% of the urinary oxalate [9] but can be as high as ~50%, as oxalate is neither stored nor further metabolized inside the body [2]. Different studies have shown that foods rich in oxalate cause a transient state of hyperoxaluria, therefore difficult sometimes to detect in 24 h urinary samples [4, 30] Another mechanism for hyperoxaluria is fat malabsorption, also known as enteric hyperoxaluria. It can arise for two different reasons: (a) greater access of the mucous membrane in the intestine to oxalate caused by greater numbers of dihydroxy bile acids such as taurocholic and glycocholic acid and (b) interaction of fatty acids with calcium present in the lumen, augmenting the quantity of soluble oxalate when few insoluble CaOx complexes are formed [31]. This condition has been shown to be linked with bypass surgeries of small distal bowel or resections and other pathophysiological disorders in which grave steatorrhea occurs, for example, in pancreatic insufficiency and celiac spruce in both children and adults. Furthermore, patients who have had jejunoileal bypass surgery also tend to have higher rate of occurrence of enteric hyperoxaluria. Additional reasons for malabsorption include biliary obstruction, overgrowth of bacteria, and blind loop syndrome [31].

## 2. Oxalate and Calcium Oxalate Induced Injury

Studies have shown that oxalate and calcium oxalate cause renal injury leading to inflammation and other pathophysiological conditions in the kidneys [32–35]. Oxalate levels in the urine crosses the supersaturation limits, causing crystallization of CaOx, calcium oxalate monohydrate (COM) deposition in the renal cells and tissues that leads to damage that ultimately results in end-stage renal failure [35]. Many studies have shown that oxalate and CaOx crystals lead to death of cells in *in vitro *analyses [32, 36, 37].

Oxalate ions are generated in the liver by glyoxalate metabolism, but due to low solubility they are carried at low concentrations in the plasma membrane [38]. Previous studies have shown that oxalate is quickly taken up by proximal tubule cells and high concentration of oxalate can be excreted in urine by the secretary pathway [39, 40]. The major pathway of oxalate excretion from the body is via urinary excretion; however, a study in rats has shown that large quantities of oxalate can also be removed by the gastrointestinal system when there is kidney failure [41].

It is now well known that CaOx crystals cause injury to cells and tissues by causing damage to cell membranes, production of lipid mediators (prostaglandins, leukotrienes), and excessive production of reactive oxygen species, all of which lead to an imbalance between oxidants and antioxidants, with malfunctioning of mitochondria [42, 43]. Studies have shown that CaOx crystals induce the phosphatidylserine imbalance in the membrane and greater production of ceramide, signals of cell death [42–44]. CaOx also causes hemolysis of red blood cells [45] and CaOx crystal injury may also be due to abundant release of ROS and other free radicals produced from molecular oxygen which ultimately lead to oxidative stress. Our review provides an insight on oxalate- and CaOX-induced renal injury due to different types of ROS produced by numerous enzyme complexes and mitochondria with special focus on NADPH oxidases leading to oxidative stress.

## 3. Reactive Oxygen Species (ROS) and Oxidative Stress

**Figure 1:**
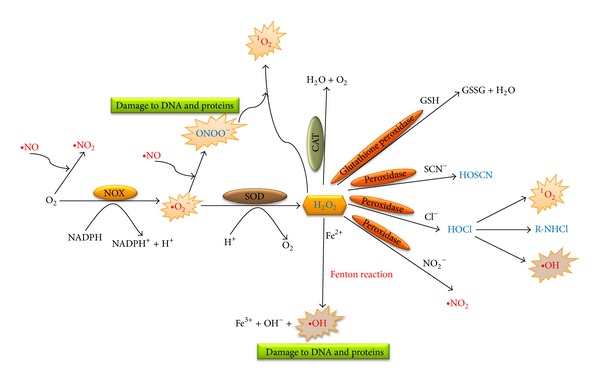
Production of ROS and different reactions. ROS with 1 free electron are shown in red and 2 free electrons are shown in blue. ROS, when produced in excess, cause damage to different components of the cell. Excess production of hydrogen peroxide (H_2_O_2_) and peroxynitrite (ONOO^−^) leads to the production of singlet oxygen (^1^O_2_). The other radicals shown in the figure are superoxide (•O_2_^−^), nitric oxide (•NO), nitrogen dioxide (•NO_2_), hydroxyl radical (•OH), glutathione (GSH), glutathione disulphide (GSSG), thiocyanate (SCN^−^), hypothiocyanous acid (HOSCN), hypochlorous acid (HOCl), and chroramine (R-NHCl). Figure modified from [1, 46].

*Reactive oxygen species* are chemically reactive molecules and free radicals generated from molecular oxygen that, if produced in excess, cause damage to tissues and different components of the cells. Yet, if produced in physiological balance, ROS have been shown to play a principle role in normal cell signal transduction pathways, including apoptosis, gene expression, and activation of different cell signaling cascades. They are produced by different constitutively active oxidases such as NADPH oxidase, xanthine oxidase, lipoxygenase, cyclooxygenase, hemeoxygenase, and in the electron transport chain of mitochondria during cellular respiration [1, 46]. Major cellular ROS include the superoxide anion (O_2_^−•^), nitric oxide radical (NO^•^), hydroxyl radical (OH^•^), and hydrogen peroxide (H_2_O_2_), all of which are produced by different signaling pathways [1, 46]. The superoxide anion, precursor of the more powerful and complex oxidants, is mainly produced by the respiratory burst of phagocytes which is regarded as the most significant free-radical generator *in vivo *[47]. These ROS may react with chemicals and enzymes to generate additional oxidative species or become ineffective by nonenzymatic and enzymatic intercellular and intracellular reactions [48]. O_2_^−•^ reacts with nitric oxide (NO) to produce peroxynitrite (ONOO^−^) which is a highly reactive and toxic nitrogen-containing species which nitrates proteins causing nitrative stress, augment platelet aggregation and vasoconstriction of the blood vessels [49]. Due to this reaction, there is diminished bioavailability of NO, a cell-to-cell messenger, and this causes beneficial effects such as decreasing blood pressure [50]. Superoxide is highly reactive, has a short half-life, cannot cross the cell membrane, and is therefore acted on by the scavenging enzyme, superoxide dismutase (SOD), which converts it to hydrogen peroxide (H_2_O_2_). Hydrogen peroxide is more stable as compared to superoxide and it diffuses though the lipid bilayer. Hydrogen peroxide (H_2_O_2_) is further acted on by another scavenging enzyme, catalase (CAT), which neutralizes it to water and oxygen (Figure 1). In a metal catalyzed reaction, called the Haber-Weiss reaction, hydrogen peroxide yields a short-lived, short-ranged, and more reactive hydroxyl radical. Also, in the presence of Fe^2+^, a highly reactive hydroxyl radical (OH^•^), is formed (Fenton reaction). Hydrogen peroxide, after oxidation by myeloperoxidase, gives rise to another extremely reactive oxygen species, hypochlorous acid (HOCl). Hypochlorous acid is a powerful oxidizing agent which is known to alter lipid structure and function, other membranous components of the cells and proteoglycans. It acts on thiol groups of membranous proteins and is known to cause chlorinative stress [49]. Studies have shown that hypochlorous acid, along with hypobromous acid (HOBr), and hypothiocyanous acids (HOSCN) have a role in antimicrobial defense by neutrophils [48, 51]. These reactive oxygen species under normal conditions function as mediators in different cell signaling and regulatory pathways involving growth and proliferation, activation or inhibition of different molecules and in regulating different transcriptional activities. Signaling molecules that are controlled by these ROS include phosphatases, Ras, phospholipases, calcium signals, serine/theonine kinases and protein tyrosine kinases. ROS also regulate different nuclear factors such as nuclear factor-*κ*B (NF*κ*B), transcription factor activation protein-1 (AP-1), and different genes such as *c-myc, c-fos*, and *c-jun* (1). ROS are also involved in initiation and implementation of programmed cell death (apoptosis). Under normal conditions, these ROS and reactive nitrogen species (RNS) are present at equilibrium with other antioxidants and are only generated when required and then vigorously removed by various scavenging enzymes and antioxidants. They play significant regulatory roles in various physiological processes, including innate immunity, modulation of redox-dependant signaling pathways, and as cofactors in the production of hormones.

ROS, when overproduced, can lead to oxidative stress. The majority of cells respond by increasing the levels of intracellular levels of antioxidants, but an excess of oxidants within a biological system leads to a change in the redox state, towards one that is more oxidizing [52, 53]. Oxidative stress or abundance of ROS causes permanent damage to macromolecules and also causes interference in the important redox-dependant signaling processes [54]. Oxidative stress causes disruption of the nitric oxide (NO) signaling pathway [55]. NO has anti-inflammatory and vasodilator functions, but under excessive ROS, gets converted to peroxynitrite [56, 57], a powerful oxidant that causes oxidation of small-molecule antioxidants such as glutathione, cysteine, and tetrahydrobiopterin [58]. Limited presence of tetrahydrobiopterin leads to uncoupling of endothelial nitric oxide synthase (eNOS), which in turn changes this enzyme from an NO-producing, vasoprotective enzyme to a superoxide-producing, oxidative stress enzyme [59, 60]. Peroxynitrite is very harmful and can hinder the activity or totally deactivate useful antioxidant enzymes such as superoxide dismutase, glutaredoxin, and glutathione reductase [58]. Peroxynitrite causes oxidation of the zinc thiolate center of NO synthase resulting in decreased formation of NO [61]. Decrease in NO can lead to increase in inflammation and remodeling of different biomolecules. Research has shown that ROS cause change in confirmation due to oxidation of proteins, such as kinases and phosphatases, and activation of nuclear factor-*κ*B (NF*κ*B) which play important roles in the regulation of immune response to infection [62]. NF*κ*B is mainly involved in transcription where incorrect regulation can lead to inflammation, cancer, and autoimmune diseases. Activation of NF*κ*B also leads to expression of adhesion molecules such as ICAM-1 (intercellular cell adhesion molecule-1), VCAM-1 (vascular cell adhesion molecule-1), and E selectin on the endothelium [63]. NF*κ*B activation also leads to proliferation and migration of vascular smooth muscle cells [64]. In this regard, ROS are also known to excite different cytosolic molecular complexes known as inflammasomes that have enzymatic activity mediated by the activation of caspase-1. Inflamasomes are involved in maturation and cleavage of cytokines such as IL-1*β* which is involved in inflammatory response [65]. 

There are a variety of markers in the kidneys which increase during oxidative stress. These include an increase in renal excretion of lipid peroxidation markers, but this increase in renal excretion is not a proof of increased ROS. Research has shown that there is greater excretion of 8-Isoprostane, PGF_2*α*_, and malondialdehyde (MDA) by long-time infusion of ANG II in rats [66, 67]. Also one study has shown that animals secrete significantly higher amounts of thiobarbituric acid reactive substances (TBARS) in the urine, generated as a byproduct of lipid peroxidation and an indication of oxidative stress in the kidneys [68]. The presence of *α*-glutathione S-transferase (*α*-GST) in the urine of animals was also shown to be an indication of oxidative stress in the kidneys [68]. Oxidative stress is due to excessive production of ROS or reduction in antioxidants leading to production of free radicals that are injurious to all components of the cell including proteins, lipids, and DNA. Oxidative stress also leads to interruption in the normal signaling processes. **β*-galactosidase *(GAL) and N-*acetyl*-**β*-glucosaminidase *(NAG), both markers of renal epithelial injury, also showed increased excretion in the urine [69]. Previous research has also shown greater urinary MDA, plasma MDA, and urinary NAG activity but diminished glutathione (GSH), cellular glutathione peroxidase (cGPx), protein thiol, and vitamin E activity observed in patients diagnosed with kidney stones which showed decreased urinary MDA, plasma MDA, and increased vitamin E after supplementation with potassium citrate (60 mEq/day for 1 month) [1]. There is also an increase in the ROS-dependant products such as an increase in the renal nitrotyrosine immunoreactivity in kidneys of SHR [70], 2k, 1C rats [71]. Also, it is possible to take direct measurements of ROS such as superoxide production in the medulla [72] and the production of H_2_O_2_ by an ANG type 1 receptor-dependant mechanism in rats which helps us estimate the degree of oxidative imbalance in the kidneys [73]. These abovementioned markers provide an estimate of OS and renal injury but further studies and validation of all markers of OS would greatly augment our understanding of the role OS plays in causing renal injury.

## 4. Isoforms of NADPH Oxidase

**Figure 2:**
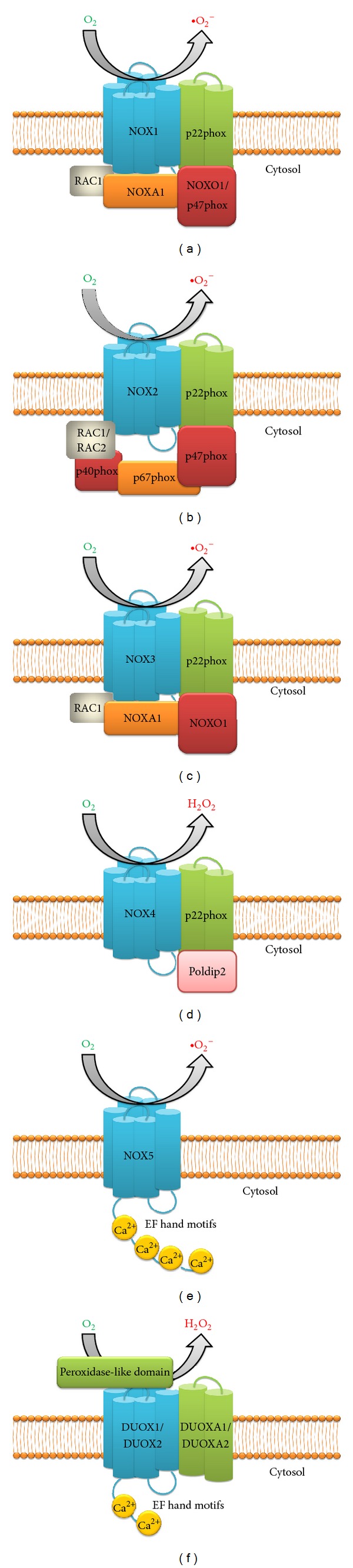
Seven different NOX isoforms-NADPH oxidase complexes. NOX isoform expression is relatively regulated at different transcriptional, post-transcriptional and translational levels under certain pathophysiological conditions. Most of the NOX isoforms have structural similarities to NOX2, with maximum in NOX3. NOX4 is most abundant in the kidneys in various kinds of cells. NOX4 is known to be constitutively active and do not require any subunits. NOX5 is directly activated by calcium. The core subunits of all the complexes (NOX1-NOX5, DUOX1/DUOX2) are shown in blue; their membrane bound subunits (p22phox, DUOXA1 and DUOXA2) are shown in green; the cytosolic subunits which acts as organizers (p40phox, NOXO1 and p47phox) are shown in red; activator subunits of NADPH oxidase complexes present in the cytosol (p67phox and NOXA1) are shown in orange; small GTPases (RAC1 and RAC2) are shown in grey; EF hand motifs are shown in yellow which bind with calcium to regulate the activity of NOX5, DUOX1 and DUOX2 (see text for details).

To date, seven different isoforms of NADPH oxidase have been described. These are NADPH oxidase 1 (NOX1), NADPH oxidase 2 (NOX2), NADPH oxidase 3 (NOX3), NADPH oxidase 4 (NOX4), NADPH oxidase 5 (NOX5), Dual oxidase 1 (DUOX1), and Dual oxidase 2 (DUOX2). These isoforms are comprised of different core catalytic subunits: p22phox, p47phox, p67phox, p40phox, DUOX activator 1 (DUOXA1), DUOX activator 2 (DUOXA2), NOX activator 1 (NOXA1), and NOX organizer 1 (NOXO1) (Figure 2). These regulatory subunits are involved in different functions. While p22phox, DUOXA1, and DUOXA2 are responsible for the growth and expression of the NOX and DUOX core units in biological membranes, P67phox, and NOXA1 are involved in enzyme activation and p40phox, p47phox, and NOXO1 in the spatial organization of different subunits of the enzyme [74]. RAC1 and RAC2 (small GTPases) may also be involved in the activation in some isoforms of NADPH oxidase, *per se*. Most of the isoforms generate superoxide except NOX4, DUOX1, and DUOX2 oxidases which directly generate H_2_O_2_ [75, 76]. NOX2 or gp91phox (91-kDa glycoprotein), previously known as mitogenic oxidase 1 (mox-1), along with p22phox (22-kDa protein) forms the two components of flavocytochrome b_558_, a heterodimeric integral membrane protein [77]. NOX2 is a catalytic subunit which produces superoxide and is a protein which consists of six transmembrane domains with cytosolic C- and N-terminus [78]. Studies have shown that NOX2 has highest structural similarity with NOX3 (58%), followed by NOX1 (56%). NOX4 and NOX5 are remotely associated with NOX2 showing around 37% and 30% resemblance, respectively [77]. NOX5 has more structural similarity with the DUOX's subunits as they all have EF hand motifs (calcium-binding motifs) [77]. NOX1 isoform has been shown to be concerned with redox-dependent cell signaling and regulation of gene expression [79] and is mainly expressed in the colon epithelial cells [80]. However, other studies have shown NOX1 to be present in vascular smooth muscle cells (VSMC), sinusoidal endothelial cells, uterus, prostate, osteoclasts, placenta, retinal pericytes, and microglia [78]. NOX2 expression is well established in the phagocytes [81–83] but has also been observed in nonphagocytic cells such as neurons, hematopoietic stem cells, smooth muscle cells, endothelium, cardiomyocytes, skeletal muscle cells, hepatocytes, and neutrophils [78, 81]. NOX3 isoform is known to be significantly expressed in the inner ear, fetal kidney, brain, and skull [84], while NOX3 has been shown to be favorably localized in cochlear and vestibular epithelial cells as well as spiral ganglion [78]. NOX4 isoform or renal NADPH oxidase (RENOX) is known to be highly expressed in the kidneys and is found in different cell types including neurons, smooth muscle cells, adipocytes, keratinocytes, hematopoietic stem cells, melanoma cells, fibroblasts, osteoclasts, and endothelial cells. NOX4 is the predominant isoform in the endothelial cells [85–88]. NOX5 isoform has been found in different parts of the body such as testis, vascular smooth muscles, ovaries, lymph nodes, myometrium, pancreas, spleen, and prostrate [89–91]. NOX5 has been involved in cell growth and thus far 5 subtypes, namely, NOX5*α*, *β*, *δ*, *γ*, and *ε*, have been found [89–92]. The other isoforms of NOX, DOUX1, and DOUX2 originally identified as thyroid oxidases have been extensively found in the thyroid [93], but also in prostate gland and airway epithelial cells. DUOX1 is expressed in bronchial and tracheal airway epithelial cells, whereas DUOX2 is found in epithelial cells of salivary glands, stomach, and brush border cells of various rectal glands such as caecum and sigmoidal colon [94]. All of these NOX isoforms play a significant role in the generation of ROS and oxidative stress. These enzymes are involved in many pathophysiological processes that are very crucial for different functions such as cellular signaling, regulation of gene expression, and cell differentiation.

## 5. NADPH Oxidases in the Kidney

**Figure 3:**
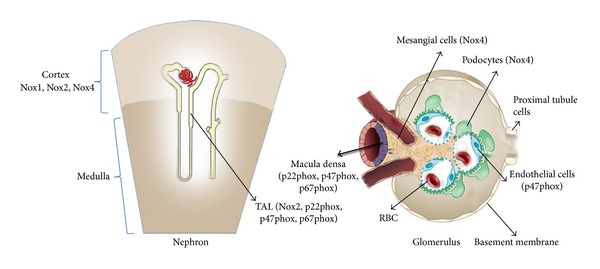
Different isoforms of NADPH oxidase complex present in different parts of the kidneys. The Nox isoforms expressed in the cortex and medulla as shown in the nephron and different cellular populations in the glomerulus (see text for details).

Research has shown that NADPH oxidase in the kidneys may have a specific pathophysiological role; thus, it is present in different cellular compartments of the kidneys (Figure 3). The mammalian kidney consists of different cellular populations including mesangial cells, fenestrated endothelial cells, tubular epithelial cells of the proximal and distal nephron segments, glomerular podocytes, dendritic cells, and the cortical fibroblasts [95, 96]. Previous research has shown that the main supplier of ROS in the form of superoxide O_2_^−•^ in the renal cortex is NADPH oxidase, whereas in the renal medulla xanthine oxidase also makes similar contribution to O_2_^−•^ generation along with NADPH oxidase [97]. Different subunits of the NADPH oxidase have also been shown to be abundantly present in the macula densa (MD), thick ascending loop of henle (TAL), interstitial cells, blood vessels, glomeruli, and tubules in the kidneys of spontaneously hypertensive rats (SHR) [98]. Previous studies in the human glomerular mesangial cells (HMC) have recognized *α* and *β* subunits of cytochrome b_558_ and the 45-kDa flavoprotein [99]. Human glomerular mesangial cells produce ROS such as superoxide and express different NADPH oxidase subunits like p22phox, p67phox, and p47phox [100] and Nox4 [101, 102]. Studies have shown that the thick ascending loop of henle (TAL) in the outer medullary region expresses different NADPH oxidase subunits such as p40phox, p47phox, p22phox, and Nox2 [103]. Podocytes or visceral epithelial cells present around the capillaries of the glomerulus in the kidneys play a significant role in the functioning of the glomerular capillary wall. Research has shown the production of ROS in the cultured human podocytes and ROS was generated by NADPH oxidase and different subunits of NADPH oxidase such as p67phox, p47phox, Nox2, and p22phox were expressed in the podocytes, present in the glomerulus of the kidneys [104]. Nox4 has been shown as the most common Nox isoform to be expressed in the kidney epithelial cells [105, 106] and is distributed in the microvasculature, glomeruli, mesangial cells, and nephron segments [102, 105, 107]. Nox1, Nox2, and Nox4 have also been shown to be expressed in the renal cortex [66, 108]. Chabrashvili et al. have shown the expression of p67phox, p22phox, and p47phox in the renal cortex [66, 98]. The same group compartmentalized the NADPH oxidase subunits in the renal cortex to macula densa, specific nephron segments in the TAL, cortical and medullary ducts, distal convoluted tubule, renal microvasculature and glomeruli [66, 98]. P47phox which is also known as neutrophil cytosol factor-1 (Ncf-1) was found to be present in the endothelium and glomerular podocytes and p22phox subunit in the renal interstitial fibroblasts [98]. These studies give us an insight on the role of different NADPH oxidase subunits found and expressed in various subcompartments of the kidneys, making NADPH oxidase complex as one of the most important contributor of oxidative stress in the kidneys.

## 6. Regulation of NADPH Oxidase (NOX Enzymes) Expression in the Kidney

It is now well accepted that a significant amount of ROS production in mammalian cells is derived from the NADPH oxidase (NOX) of phagocytes (Phox), especially neutrophils and macrophages that catalyze the respiratory burst (i.e., the production of large number of ROS and utilization of large amounts of O_2_) [109]. Normally, the NADPH oxidase is nonfunctional but can be activated quickly when a cell comes in contact with different inflammatory biomolecules or microorganisms resulting in generation of ROS apart from mitochondrial production. Cytosolic NADPH oxidase is the electron donor for all the NADPH oxidase isoforms with molecular oxygen acting as the final electron acceptor. The electron transfer to molecular oxygen results in the release of superoxide from the oxidase enzyme in NOX1, NOX2, and NOX5 isoforms [110]. The other NOX isoforms such as NOX4, DUOX1, and DUOX2 oxidases do not directly release superoxide anion as their primary ROS; instead, they release hydrogen peroxide [75, 76]. The NADPH oxidase complex consists of the membrane subunits Nox2 (gp91phox) and p22phox along with the regulatory cytosolic subunits p67phox, p47phox, p40phox, and the small GTPases protein, RAC [111]. 

Research has shown that NADPH oxidase is activated by Ang II infusion in the rat kidneys leading to increased expression of p22phox and Nox1 in the renal cortex with concomitant reduction in the presence of Nox4 and SOD [66]. Also, high salt intake increased oxidative stress by increasing the expression of NOX2 and p47phox subunits and decreased SOD expression [108]. The prolonged effect of Angiotensin in the kidney has been shown to cause the activation of NADPH oxidase, enhanced expression of p22phox, and decrease in the scavenging enzyme SOD leading to renal cortical hypoxia, renal vasoconstriction, and hypertension [67]. The Nox1 subunit has been shown to be upregulated in the rat-cultured vascular smooth muscle cells (VSMC) by PDGF, Ang II, and serum [112], whereas research has shown downregulation of Nox4 in the kidney cortex by the infusion of Ang II [108]. Ang II has been shown to upregulate p67phox expression in rabbit periadventitial fibroblasts [113] and the mouse aorta [114]. These research findings provide a brief insight on the role of angiotensin on the different subunits of NADPH oxidase and the regulation of expression of NADPH oxidase in the kidneys.

## 7. Antioxidants for Renal Treatment

Antioxidants have been shown to reduce oxidative stress. Treating the kidneys with vitamin E (*α*-tocopherol) along with mannitol removed the chances of deposition of CaOx crystals in rat kidneys injected with sodium oxalate [115]. Furthermore, antioxidants such as methionine, combination of vitamin E plus selenium, and glutathione monoester subdued CaOx crystals in the hyperoxaluric rat kidneys [116–118]. However, recent studies have shown that it is not easy to remove oxidative stress with increased levels of antioxidants such as vitamin E in clinical trials [119–121]. These disparate observations cannot be regarded as proof against antioxidants as several clinical trials involved high risk patients in which the end-stage renal disease was quite advanced and doses of vitamin E differed greatly between trials. Antioxidant concentration is very critical in controlling oxidative stress because of the very high rate constants of the reactions between ROS and other molecules such as NO, certain amino acids, and nucleic acids. The reaction between NO and ROS happens at a rate of 1.9 × 10^10^ M^−1^ S^−1^ which is 6 times faster in magnitude than the reaction between superoxide and vitamin E [122, 123]. Vitamin E in the body also faces a highly oxidizing environment one that can lead to rapid removal of reduced forms of antioxidants. It would seem that the best approach for reducing oxidative stress is by targeting the enzyme responsible for the generation of ROS, perhaps targeting NADPH oxidase by use of inhibitors of NADPH oxidase. 

## 8. NADPH Oxidase Inhibitors

Identification of NADPH oxidase inhibitors is an ongoing active field, focused primarily on substances that obstruct a specific NADPH oxidase from catalyzing production of superoxide. NADPH oxidase inhibitors act through interference in the assembly of the NADPH oxidase complex by interacting with their subunits, blocking electron transfer decreasing production of ROS [124].  Table 1 lists a number of chemicals that alleviate oxidative stress through inhibiting ROS production by NADPH oxidases. In addition, Table 1 also describes the mode of action and pharmacologic effects of different peptide and nonpeptide inhibitors. These chemicals include, but are not limited to, Apocynin, diphenyleneiodonium chloride (DPI), pefabloc, proline-arginine rich antimicrobial peptide (PR-39), and new peptide inhibitors that have been developed to particularly target NADPH oxidases, such as gp91 ds-tat and novel nonpeptide VAS2870 [125]. The two most studied NADPH oxidase inhibitors are Apocynin and DPI. Apocynin, also known as 4-hydroxy-3-methoxy acetophenone or acetovanillone, is the best known inhibitor of NADPH oxidase to date. It was extracted from the roots of *Apocynum cannabinum* by Schmiedeberg in 1883 [126] and found to block the association of p47phox with membrane-bound p22phox subunit of the NADPH oxidase complex in leukocytes, monocytes, and endothelial cells and is also a scavenger of H_2_O_2_ [127]. At high concentration, it was shown to block Nox4, and Nox5 [128], making it more effective against Nox2, Nox4, and Nox5 dependant NADPH oxidase-dependant activity. Apocynin has been shown to reverse activation of the NADPH oxidase system in rat kidneys induced by hydroxyl-l-proline (HLP) treatment even in the face of high levels of hyperoxaluria, revealing the role of Apocynin as an inhibitor as well as having antioxidant inductive activities [129].

The most frequently used inhibitor of NADPH oxidase is diphenyleneiodonium chloride (DPI), also known as dibenziodolium chloride. Its mode of action is by taking electrons from electron transporter and creating a radical which blocks the appropriate transporter of electrons through a covalent binding step [124]. Regarding NOX isoforms, the action of DPI appears to be nonspecific towards any isoform and it partially or completely inhibits different types of enzymes such as iNOS, xanthine oxidase, and NADPH oxidase [124].

## 9. Summary

In this review, we talk about renal injury caused by oxalate and calcium oxalate crystals due to hyperoxaluria. Oxalate and calcium oxalate can lead to renal injury due to disruption of membranes, ROS-induced oxidative stress, and mitochondrial dysfunction. We put the main emphasis on oxidative stress caused by ROS produced by different isoforms of NADPH oxidase as it has been found that these different isoforms of NADPH oxidase are one of the most important contributors of ROS and oxidative stress produced in the different subcellular localizations of the kidneys. These NADPH oxidase complexes play a crucial role in host defense, various signaling pathways leading to regulation of gene expression, and protein functions under normal conditions of oxidative balance. When this oxidative balance is disturbed due to environmental and/or physiological processes, the potential of the NADPH oxidases in inducing injury to both microorganisms and cells makes regulation essential, not only through normal physiological activities, but also exogenous inhibitors. Chemicals that inhibit generation of ROS provide considerable benefits over general antioxidants such as vitamin E, which appears to be less efficient due to various properties, including decreased bioavailability. It would seem, therefore, that in order to reduce the function and downstream effects of NADPH oxidase, a main focus should be on blocking the assembly of NADPH oxidase subunits. Various peptide and non-peptide inhibitors are known which mainly operate by disrupting the association of NADPH oxidase complex assembly. Special focus should be on targeting the organizer subunit, that is, p47phox or the NOXO1 subunits. Other molecular subunits for therapy may be the activator subunits such as p67phox and NOXA1 along with Rac. Thus, the main focus should be to develop an inhibitor with increased efficiency and specificity of binding with the protein subunit. Comprehensive studies are needed on the molecular subunit structures to be targeted and their effects on interactions with other subunits present downstream in the NADPH oxidase complex.

## Figures and Tables

**Table 1:** Inhibitors of NADPH oxidase.

Name	Mode of action	Pharmacological effects	References
Apocynin	NADPH oxidase complex assembly inhibitor: inhibits binding of p47phox with membrane bound p22phox	Scavenger of H_2_O_2_	[130–132]
DPI	Inhibitor of flavoprotein, takes electrons from FAD and prevents electron flow through the flavocytochrome channel	Inhibitor of NADH-ubiquinone oxidoreductase, NADH dehydrogenase, xanthine oxidase, cytochrome p450 oxidoreductase, NOS, and bacterial nicotine oxidase	[133–139]
AEBSF	Inhibits association of NOX2 subunit with p47phox. Prevents binding of p47phox and p67phox with cytochrome b559	Irreversible serine protease inhibitor	[140]
Plumbagin	Inhibits O_2_^−•^ production in various cell lines expressing NOX4 oxidase; unknown mechanism	Naphthoquinone structure may confer ROS-scavenging effects	[141, 142]
PR-39	Inhibits p47phox from binding to p22phox subunit by cohering to SH3 domains of p47pphox	Non selective for NADPH oxidase	[143]
S17834	Flavonoid derivative proposed to directly inhibit NADPH oxidase activity, although the mechanism is undefined	None	[144]
VAS2870	Undefined mechanism: inhibits NADPH oxidase activity in NOX2 oxidase-containing HL-60 cell line and in vascular endothelial cells containing NOX2 and NOX4 oxidases; does not scavenge O_2_^−•^	None	[145, 146]
Gp91dstat	Oxidase assembly inhibitor: inhibits association of Nox2 with p47phox. Does not scavenge superoxide generated by cell-free systems	None	[147, 148]
Statins	Decrease superoxide production by inhibiting synthesis of farnesylpyrophosphate and geranylgeranylpyrophosphate which are crucial for membrane attachment of Rac and NADPH oxidase assembly. May also decrease p22phox and Nox1 expression. Likely to influence Nox1 and Nox2 activities	HMG-CoA reductase inhibitor. Decreases AT1 receptor expression; increases eNOS expression, most effective for treating cardiovascular disease with questionable benefit in those without previous CVD but with elevated cholesterol levels	[149, 150]
AT1 receptor antagonists	Decrease Ang II-dependent activation of NADPH oxidase via AT1 receptors. Unlikely to display Nox selectivity as Ang II stimulates Nox1 and Nox4 oxidases	None. Controlling high blood pressure	[151]
Nebivolol	Inhibits membrane association and also interaction of p67phox and Rac and decreases oxidase expression. Inhibits Nox1-dependent superoxide production	*β*-adrenoceptor blocker, used in treatment of hypertension	[152–155]
Gliotoxin	A fungal metabolite, thiol-modifying toxin thought to inhibit phosphorylation of p47phox by preventing PKC colocalization with p47phox. Also, inhibits electron transport through the flavocytochrome before oxidase activation. Low potency for blocking Nox4	Stimulation of cGMP release. Cytoskeletal reorganization. Disrupts the mitochondrial membrane potential, possesses immunosuppressive properties, anti-inflammatory activity.	[75, 156–160]
Clostridium difficile toxin B	Glycosylation of threonine-35 on Rac, which modifies GTPases activity	Likely to inhibit all Rac-dependent protein activity. High toxicity, vascular permeability and inflammation	[161]
Nordihydroguaia-retic acid	Blocks H_2_O_2_ production in macrophages in response to phorbol esters and in endothelial cells in response to thrombin	Lipooxigenase inhibitor. Blocks arachidonic acid metabolism	[162–164]
SKF525A	Decreases superoxide and H_2_O_2_ production in endothelial cells	Cytochrome P450 inhibitor	[162, 165]
Metformin	Scavenges hydroxyl radicals but not superoxide. Could also inhibit PMA and Ang II-dependent ROS production from NADPH oxidase. However, this is likely to be due to inhibition of PKC activity	Antihyperglycemic agent. PKC inhibitor	[166–168]
Sildenafil-citrate	Inhibitor of endothelial superoxide production and gp91phox expression	Inhibits phosphodiesterase type 5. Nonselective and in direct inhibitor for NADPH oxidase isoforms. Have been shown to influence immune system due to changes in gp91phox expression	[169–172]
Bilirubin	Inhibitor of superoxide production. No effect on Nox2, p22phox and p47phox but may reduce p47phox phosphorylation	ROS scavenger	[173–175]
Minocycline	Downregulates p67phox expression. Inhibitor of superoxide generation in microglia and dopaminergic neurons in response to stimuli such as thrombin	Antibiotic	[176, 177]
Perhexiline	Inhibits superoxide production in intact neutrophils stimulated by fMLP or PMA. Mechanism unknown	Efficient antianginal agent that blocks carnitine-palmitoyl-transferase	[178, 179]
Roxithromycin	Inhibits superoxide generated by intact neutrophils activated by fMPL or PMA but not by cell lysates. No effect on PKC-dependent phosphorylation. May inhibit translocation of p47phox and/or p67phox	Macrolide antibiotic. Inhibit RNA-dependent protein synthesis. Efficient in blocking cytochrome P450	[180–182]
Taurine chloramines	Inhibits translocation of p47phox and p67phox to the membrane. Also inhibits phosphorylation of p47phox. Reversible inhibition of PMA-dependent superoxide anion production in human neutrophils	Blocks inducible NOS in alveolar macrophages	[183, 184]
Resveratrol	Reduces superoxide generation in intact macrophages and homogenates. Does not scavenge superoxide in cell-free systems	Inhibitor of PKC	[185–187]
Curcurmin	Reduces superoxide production in intact macrophages and homogenates. Does not scavenge superoxide in cell-free systems	Irreversible inhibitor of thioredoxin reductase via alkylation of cysteine residues	[185, 188]
Nitrolinoleate	Nitrated lipid which blocks PMA- and FMLP-dependent superoxide generation and degranulation in human neutrophils by enhancing cAMP but not cGMP levels	Also linked with increasing cAMP vasorelaxation	[189, 190]
Mycophenolate acid	Fungal derivative that blocks endothelial and neutrophil-derived superoxide by reducing Rac levels. Does not alter mRNA levels of Nox2, Nox4, and p47phox	Efficient inhibitor of inosine monophosphate dehydrogenase associated with purine synthesis in B and T lymphocytes	[191, 192]
GK-136901	Well known NOX1 and NOX4 oxidase inhibitor. Unknown mechanism, but structural similarity with NADPH suggests that it may act as a competitive substrate inhibitor of this enzyme	None	[193, 194]
ML171	Phenothiazine compound with selectivity for NOX1 oxidase (IC_50_ of 0.25 *μ*M) over other NADPH oxidases (IC_50_ > 3 *μ*M). Does not scavenge oxygen radicals generated by xanthine oxidase activity	None	[195]
Mastoparan	Inhibits superoxide production by neutrophil lysates most likely via interaction with N-terminal of p67phox	An amphiphilic cationic tetradecapeptide isolated from wasp venom. Has affinity towards SH3 domains. Also interact with G-proteins	[196–199]
Ghrelin	Blocks superoxide production by thoracic aorta most probably via release of NO. Does not scavenge superoxide	Capable of releasing growth hormone releasing peptide. Stimulates gastric acid secretion	[200–202]
Alpha tocopherol	Inhibitor of p67phox-p47phox translocation and p47phox phosphorylation in monocytes, neutrophils and microglial cells. This effect is likely to be due to PKC inhibition	ROS scavenger	[203–206]
Benzylisothiocyanate	Concentration-dependent. Inhibits TPA-induced superoxide production in a human leukemia cell line. Does not affect PKC activity and p47phox translocation. Mechanism may involve covalent cysteine modification of the NADPH oxidase	May inhibit NO, PGE2 and TNF-*α* production. Also known to cause apoptosis via induction of Bak and Bax proteins	[207–209]
Probucol	Known to reduce superoxide production in rabbit aorta, by down-regulation of p22phox	Free radical scavenger	[54, 210–213]
Nox2ds-tat	Oxidase assembly inhibitor: inhibits association of NOX2 subunit with p47phox. Does not scavenge O_2_^−•^ generated by cell-free systems	None	[148, 214]
VAS3947	Triazolopyrimidine that decreased ROS production in several cell lines with low micro molar efficiency, irrespective of the specific isoforms expressed; showed no inhibitory effects against xanthine oxidase-derived ROS or eNOS activity	None	[215]

Adapted from [125, 216].

eNOS: endothelial nitric oxide synthase; IC_50_: half-maximal inhibitory concentration; Nox: NADPH oxidase; O_2_^−•^: superoxide; ROS: reactive oxygen species; SH3: Src homology 3; DPI: diphenyleneiodonium chloride; AEBSF: 4-(2-aminoethyl)-benzenesulfonyl fluoride; S178341: 4-dimethyl-2,3,5,6-triiodobenzene; VAS-2870: 3-benzyl-7-(2-benzoxazolyl) thio-1,2,3-triazolo (4,5-d) pyrimidine; SKF 525A: 2-diethylaminoethyl 2:2–diphenylvalerate hydrochloride.
